# Artificial Intelligence for Fibrosis Diagnosis in Metabolic-Dysfunction-Associated Steatotic Liver Disease: A Systematic Review

**DOI:** 10.3390/diagnostics16020261

**Published:** 2026-01-14

**Authors:** Neilson Silveira de Souza, Théo Cordeiro Veiga Vitório, Raphael Augusto de Souza, Marcos Antônio Dórea Machado, Helma Pinchemel Cotrim

**Affiliations:** 1 Faculty of Medicine of Bahia, Federal University of Bahia, Salvador 40026-010, Bahia, Brazil; neilsonsdsouza@gmail.com (N.S.d.S.); theovitorio@ufba.br (T.C.V.V.); 2 Postgraduate Program in Medicine and Health, Faculty of Medicine of Bahia, Federal University of Bahia, Salvador 40110-060, Bahia, Brazil; 3 Radtec Serviços de Física Médica LTDA, Salvador 40295-010, Bahia, Brazil; 4 Department of Radiology, Professor Edgard Santos University Hospital, Federal University of Bahia, Salvador 40110-060, Bahia, Brazil

**Keywords:** Metabolic-Dysfunction-Associated Steatotic Liver Disease, liver fibrosis, artificial intelligence, diagnosis

## Abstract

**Background/Objectives**: Artificial intelligence (AI) is an emerging technology for diagnosing liver fibrosis in Metabolic-Dysfunction-Associated Steatotic Liver Disease (MASLD), but a comprehensive synthesis of its performance is lacking. This systematic review (SR) aimed to evaluate the current evidence of AI models for diagnosing or staging liver fibrosis in patients with MASLD compared to conventional diagnostic tools. **Methods**: A comprehensive search was conducted in PubMed, Scopus, Web of Science, ScienceDirect, Embase, LILACS, IEEE Series, and Association for Computing Machinery (ACM). Primary studies applying AI to diagnose fibrosis in adults with MASLD were included. Risk of bias was assessed using the QUADAS-2 tool, and methodological reporting was evaluated according to the MINimum Information for Medical AI Reporting (MINIMAR) guideline. A narrative synthesis was performed, grouping studies by data type (clinical/laboratory vs. imaging) and summarizing diagnostic performance and clinical application. A frequency-based analysis was applied to identify the most recurrent predictive features, and an analysis of the AI architecture and application was reported. The review was registered in PROSPERO (CRD420251035919). **Results**: Twenty-one studies were included, encompassing 19,221 patients and 5237 images. Across studies, AI models consistently outperformed non-invasive scores such as Fibrosis-4 Index (FIB-4) and NAFLD Fibrosis Score (NFS). The most frequent predictive variables were identified. Despite an overall low risk of bias, methodological transparency and external validation were limited. **Conclusions**: AI is feasible for the non-invasive diagnosis of liver fibrosis in MASLD, demonstrating superior accuracy to standard clinical scores. Broader clinical application is limited by the lack of external validation and high heterogeneity among the studies. Prospective validation in diverse, multicenter cohorts is essential before AI can be integrated into routine clinical practice.

## 1. Introduction

Metabolic-Dysfunction-Associated Steatotic Liver Disease (MASLD) is characterized by hepatic steatosis identified through image methods or histology, along with at least one cardiometabolic risk criterion [1]. It is the most common chronic liver disease worldwide, affecting over 30% of the adult population, with projections exceeding 55% by 2040, largely driven by obesity and Type 2 Diabetes Mellitus (T2DM) pandemics. Notably, cardiovascular disease is the primary cause of death in these patients, highlighting MASLD as a hepatic manifestation of a systemic disorder [2]. The disease spectrum includes steatosis, steatohepatitis, a more aggressive form causing inflammation known as Metabolic-Dysfunction-Associated Steatohepatitis (MASH), fibrosis, and cirrhosis [3]. Since fibrosis is the strongest predictor of both liver-related and cardiovascular morbidity and mortality, identifying patients with clinically significant fibrosis (stage F2 or higher) is fundamental for risk stratification and therapy [4].

The detection of liver fibrosis in patients with MASLD is of substantial clinical importance, as fibrosis remains the strongest predictor of both liver-related and cardiovascular morbidity and mortality. Failure to detect fibrosis, particularly at clinically significant stages (≥F2), leads to missed opportunities for timely therapeutic intervention and risk stratification [1]. Without early identification, the disease spectrum can progress unchecked from simple steatosis to advanced cirrhosis, a stage characterized by irreversible hepatic damage and significantly higher systemic risks. Given that MASLD is a manifestation of a systemic disorder where cardiovascular complications are the primary cause of death, the absence of early fibrosis screening obscures the true prognostic window, preventing interventions that could otherwise promote liver parenchyma regeneration and reduce overall mortality [2,3].

While the liver biopsy remains the gold standard for staging fibrosis, it is an invasive and expensive procedure with significant limitations, including sampling error and interobserver variability, which makes it unsuitable for widespread screening [5]. To address this, non-invasive tests (NITs) have been developed. However, serum biomarkers such as Fibrosis-4 Index (FIB-4) and NAFLD Fibrosis Score (NFS) are only modestly accurate and yield a high proportion of indeterminate results, whereas more accurate imaging methods like elastography are not widely available in all centers [6].

Artificial intelligence (AI) is emerging as a powerful tool to address this clinical need, with the potential to transform medical diagnosis [7,8]. By analyzing vast, complex datasets, AI algorithms can identify subtle patterns to enhance diagnostic accuracy in a reproducible and automated way [9]. Initial studies are promising, with AI models demonstrating superior accuracy (AUC > 0.90) compared to traditional scores for fibrosis detection [10,11]. Ultimately, the most significant potential of AI is its ability to break the link between accuracy and cost by developing highly precise diagnostic models from routine biochemical markers, offering a scalable solution to the current screening gap [12]. Despite rapid growth and optimism, the heterogeneity of models and methodologies in the field has created a critical knowledge gap. A comprehensive systematic review is needed to synthesize existing evidence, assess its quality, and compare the performance of different AI models against both standard non-invasive tests and liver biopsy. Such synthesis is essential to inform clinical applications and guide future research.

This systematic review aims to evaluate the current evidence on the accuracy of AI for the diagnosis and staging of liver fibrosis in patients with MASLD, comparing its performance with currently used diagnostic tools. Our study provides an integrated synthesis of machine learning and deep learning models based on both clinical and imaging data, critically evaluated against the histological gold standard. We further perform a frequency-based analysis to identify the most consistently predictive biomarkers across studies, establishing a data-driven framework for model development. By assessing methodological rigor using the QUADAS-2 and MINIMAR guidelines, this review aims to offer a robust foundation for the design of clinical screening tools that are scalable, reliable, and readily integrable into routine medical practice, enabling automated and objective diagnostic support.

## 2. Materials and Methods

This systematic review was conducted in accordance with the recommendations of the Preferred Reporting Items for Systematic Reviews and Meta-Analyses (PRISMA) (Supplementary Table S1). The search was performed on 28 March 2025. The systematic review protocol was registered with PROSPERO (CRD420251035919) and was developed, reviewed and approved by all authors. No amendments were made to the information provided at registration.

The literature search was conducted across PubMed, Scopus, Web of Science, ScienceDirect, Embase, LILACS, IEEE series, and Association for Computing Machinery (ACM) databases. Studies published between 2011 and 2025 were included, with the language restricted to English. The search strategy combined descriptors related to fatty liver disease and artificial intelligence. The complete set of Boolean Search Strings is available in Supplementary Table S2.

The studies included in this review adhered to general criteria, such as (a) primary studies that applied artificial intelligence (AI), including machine learning or deep learning for the diagnosis of liver fibrosis in patients with MASLD; (b) availability of the full-text article; and (c) an adult population (age ≥ 18 years). The exclusion criteria comprised studies in pediatric populations, animal models, in vitro studies, other liver diseases, and publications that were not primary studies, such as reviews and abstracts.

In addition to these general criteria, specific criteria related to the use of AI were also evaluated, based on the MINimum Information for Medical AI Reporting (MINIMAR) guide [13]. Table 1 summarizes the inclusion and exclusion criteria adopted in this study.

### 2.1. Study Selection

Based on the PRISMA guidelines, the study selection was carried out in three stages: (1) removal of duplicates, (2) screening of titles and abstracts, and (3) full-text reading of the studies that passed the previous stages. The screening was conducted online with the assistance of the Rayyan platform by three independent reviewers.

### 2.2. Risk of Bias and Quality of Studies

The Quality Assessment of Diagnostic Accuracy Studies, version 2 (QUADAS-2) tool was used to assess the risk of bias in the included studies. Additionally, the MINimum Information for Medical AI Reporting (MINIMAR) [13] guideline was employed to analyze the AI architecture, which involves Model output, Target user, Data splitting, Gold standard, Model task, Model architecture, Features, and Missingness, as well as the evaluation process of the models presented in the studies.

### 2.3. Data Extraction and Synthesis Methods

Data extraction was performed using a Google Sheets spreadsheet. The following information was collected: author; year of publication; sample size (sum of the samples used for training, testing, and validation); type of algorithm/AI/architecture; fibrosis classification scheme based on the METAVIR scoring system (F0, F1, F2, F3 and F4) or stiffness measurement in kilopascals (kPa) in cases where elastography was used as the reference; the gold standard used for staging or diagnosing fibrosis; the data-splitting strategy; the features (variables) used to train the model; the validation method (e.g., internal or external); and diagnostic performance metrics, such as the Area Under the Receiver Operating Characteristic curve (AUROC or AUC), for the artificial intelligence as well as for other non-invasive methods used for comparison (e.g., FIB-4 or NFS). The primary performance metric evaluated was the AUROC; however, in cases where it was not available in the study, it was replaced by accuracy or the agreement rate between the AI’s diagnosis and that of pathologists. The performance results were taken from the validation group.

To identify the most significant predictive variables, a frequency analysis methodology was adopted. The analysis began by identifying studies that utilized clinical or laboratory features to train artificial intelligence models for the diagnosis of fibrosis. Following this identification, an analysis was conducted to determine which features were most frequently used across all the included articles. A descending rank-order list was then generated based on this frequency count. The final ranking includes all features that were present in at least 1/4 of the studies analyzed.

Studies were grouped for synthesis by input modality (clinical/laboratory vs. imaging), with fibrosis definitions harmonized (e.g., mapping “significant fibrosis” to ≥F2) to facilitate comparison. Results were tabulated to display model characteristics and metrics. Given the expected methodological heterogeneity precluding quantitative meta-analysis, a narrative synthesis was planned to explore potential sources of heterogeneity.

## 3. Results

**Figure 1:**
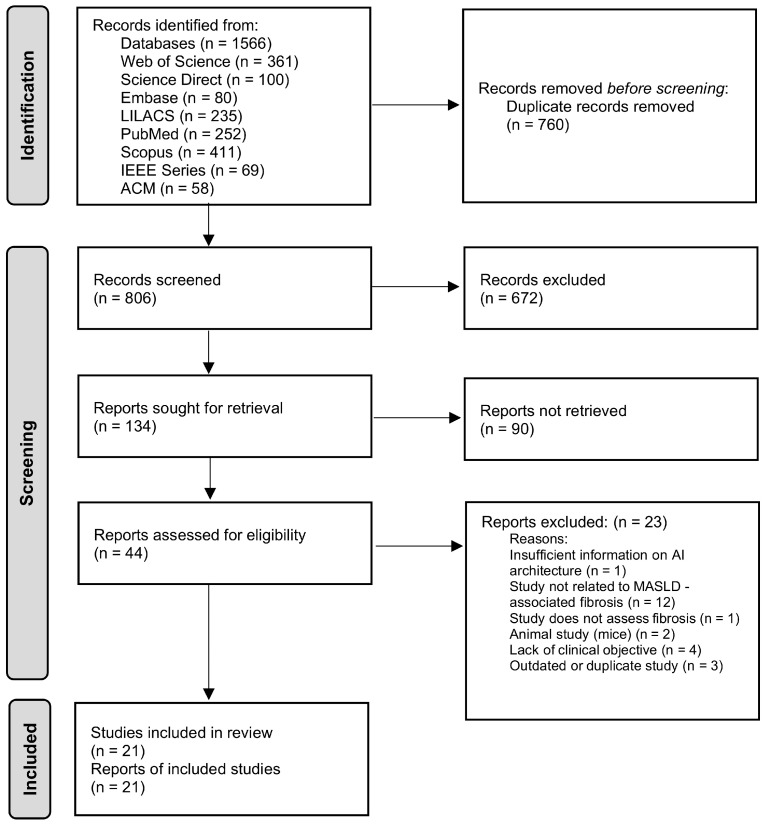
PRISMA flow diagram of the study selection for the systematic review.

The study selection process is detailed in the PRISMA flow diagram (Figure 1). A total of 21 studies met the inclusion criteria and were analyzed in this systematic review. The studies were published between 2021 and 2025. The articles were divided into two main groups based on the type of data used to train the artificial intelligence models: 15 studies that used clinical and/or laboratory [14,15,16,17,18,19,20,21,22,23,24,25,26,27,28] data and 6 studies that were based exclusively on image analysis [29,30,31,32,33,34]. In total, data from 19,221 patients and 5237 images were analyzed. The studies analyzed included diverse populations from Asia [23,26,33,34], Europe [19], and the Americas [14,24,25]. Inclusion criteria typically include patients with MASLD confirmed by biopsy [14,17,24] or, in some cases, validated non-invasive methods like elastography [18,24,30]. Exclusion criteria were consistent, removing patients with other chronic liver diseases such as significant alcohol intake (>20–30 g/day), viral hepatitis, autoimmune liver disease, or hereditary conditions [23,24,27]. Table 2 and Table 3 summarize the detailed characteristics of the included studies. In Table 2, most models based on clinical/laboratory data relied on classical machine learning (e.g., XGBoost, RF, LR, SVM), while only a small subset used shallow neural networks (ANN/FF), and no transformer-based architectures were identified. By contrast, Table 3 focuses on deep learning models applied to imaging data.

### 3.1. Risk of Bias

Quality assessment of the 21 included studies using the QUADAS-2 tool demonstrated an overall low risk of bias. Primary concerns were related to insufficient clarity in AI methodology (the index test) and the use of reference standards other than the histological gold standard, liver biopsy. Furthermore, notable heterogeneity was observed across the studies, driven by differences in patient cohorts, imaging modalities, AI architectures, and reference methods (See Supplementary Table S3 for detailed results).

### 3.2. Model Architecture

**Figure 2:**
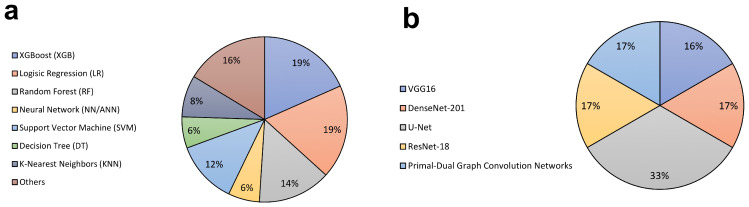
Most frequently used algorithms for the identification of liver fibrosis. This figure summarizes the algorithms applied in the reviewed studies. (**a**) This figure presents the most frequently used algorithms for fibrosis assessment based on clinical or laboratory data, highlighting methods such as XGBoost, Random Forest, and Logistic Regression. (**b**) The next figure presents the most commonly used algorithms for fibrosis assessment through imaging data, emphasizing approaches such as U-Net. Abbreviation: U-Net (U-shaped Convolutional Neural Network), DenseNet-201 (Densely Connected Convolutional Network-201), ResNet-18 (Residual Neural Network-18), VGG16 (Visual Geometry Group-16).

Among the fifteen studies trained with clinical and laboratory data [14,15,16,17,18,19,20,21,22,23,24,25,26,27,28], AI models were mainly based on machine learning, particularly ensemble methods such as Extreme Gradient Boosting (XGBoost) and Random Forest, along with Logistic Regression and Neural Networks (Table 2). For fibrosis assessment, XGBoost and Logistic Regression were the most frequently applied [14,15,16,17,20,21,23,24,25,26,27] (Figure 2). The primary goal was fibrosis classification, distinguishing early stages (F0–F1) from significant or advanced stages (≥F2 or ≥F3) [14,16,26]. Validation relied mainly on liver biopsy, though elastography was also employed in larger studies [15,19,21,25]. Features typically combined demographic, clinical, and laboratory variables.

The six studies on image-based fibrosis diagnosis used deep learning, specifically Convolutional Neural Network (CNN) architectures [29,30,31,32,34] (Table 3). Their task was classification, staging fibrosis (F0–F4) or distinguishing significant from non-significant fibrosis [32,34]. U-Net was the most frequently applied algorithm (Figure 2). Input data included histopathology slides [31,32,33,34], Magnetic Resonance Elastography (MRE) images [29], and tongue images [30]. Gold standards varied: biopsy and pathologist reports for histopathology and MRE [29,34], and SWE for tongue imaging [30]. Most studies split data into training, validation, and testing sets, with some applying *k*-fold cross-validation [31,33]. Missingness was addressed by excluding low-quality images [30]. Outputs were fibrosis stage classifications (e.g., F2, F3) [30,34]. These models were aimed at pathologists, providing automated and objective diagnostic support [29,32].

### 3.3. Model Performance

Models trained on clinical and laboratory data demonstrated strong diagnostic performance, including on external cohorts [14,16]. For instance, the ALADDIN ensemble using only laboratory data achieved an external-validation AUC of 0.717 for significant fibrosis (≥F2), outperforming FIB-4, Steatosis-Associated Fibrosis Estimator (SAFE), and LiverRisk [14]. XGBoost-based models reported by Dabbah et al. and Xiong et al. reached AUCs of 0.91 and 0.917, respectively, for advanced fibrosis, substantially exceeding FIB-4 and NFS [15,27]. Beyond classification, several studies used machine learning to identify key predictors of fibrosis progression: Suárez et al. found High-Density Lipoprotein (HDL) cholesterol, hypertension, and triglycerides to be major predictors in NASH [24], and the same group later identified platelet count as a critical predictor in MASLD patients undergoing cholecystectomy [25]. Several models were implemented as publicly available tools (NASH-Scope, FibrAIm, LiveFbr, and ALADDIN) to support clinical decision-making [14,18,22].

Six studies evaluated AI for direct image analysis to automate detection and staging of liver fibrosis (Table 3). Four focused on histopathological images from liver biopsies, aiming to match experienced pathologists and reduce subjectivity [31,32,33,34]. AutoFibroNet achieved AUCs > 0.98 on microscopy images [34], and SMART AI-PATHO showed 89.7% concordance with pathologists for non-advanced versus advanced fibrosis on conventional slides [32]. Naik et al., using multiple-instance learning on Sirius Red–stained slides, reported 78.98% accuracy and an AUC of 0.87 for distinguishing mild from severe fibrosis [31].

A comparative analysis of diagnostic performance revealed that AI models consistently outperformed traditional non-invasive tests (NITs) across the included studies. While conventional scores such as the Fibrosis-4 Index (FIB-4) and the NAFLD Fibrosis Score (NFS) generally demonstrated modest accuracy, several AI architectures, particularly those based on XGBoost and Neural Networks, achieved AUROCs exceeding 0.90 for advanced fibrosis [15,24,25,27]. However, a significant discrepancy was observed between validation methodologies; models relying solely on internal validation often reported higher and potentially optimistic AUROC values, sometimes reaching 0.92 to 0.97 [22,28]. In contrast, studies that implemented external validation, crucial for assessing generalizability, consistently reported more conservative performance metrics. This divergence likely reflects not only methodological bias related to data leakage and cohort dependency, but also the influence of batch effects and the lack of harmonization across datasets, including differences in patient populations, laboratory assays, imaging protocols, and data acquisition pipelines. Collectively, these factors may systematically inflate performance estimates in internal cohorts and partially explain the instability of reported AI performance when models are applied to independent populations. For example, the ALADDIN ensemble achieved an external validation AUC of 0.717 for significant fibrosis, which, although superior to traditional NITs in the same cohort (AUC of 0.655 for FIB-4), underscores the critical impact of external testing on performance robustness [14].

### 3.4. Model Evaluation

Internal validation was predominantly performed using the hold-out method, sometimes supplemented by cross-validation [20,24,25]. However, external validation, which is crucial for testing the generalizability of the models, was performed by a minority of studies, representing a significant limitation in the field [14,16,19,29,34]. Transparency was also limited; although some authors made their models and code available, the majority did not, hindering the reproducibility of the findings [14,23].

### 3.5. Most Frequently Used Features Across AI Models

**Figure 3:**
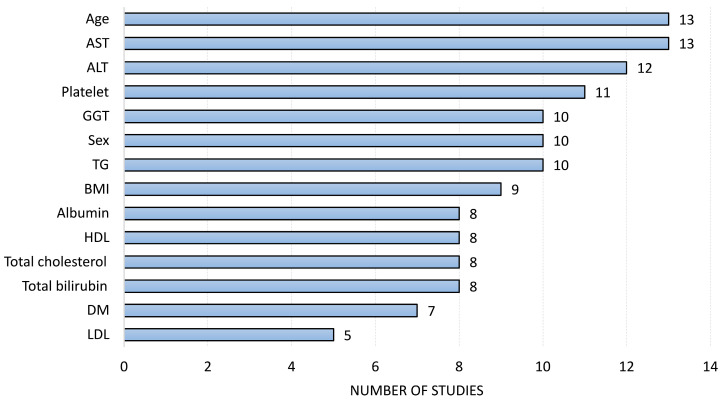
Ranking of the most frequent variables in fibrosis assessment by artificial intelligence models present in at least 1/4 of the analyzed studies. Abbreviation: AST (Aspartate Aminotransferase), ALT (Alanine Aminotransferase), GGT (Gamma-Glutamyl Transferase), TG (Triglycerides), BMI (Body Mass Index), HDL (High-Density Lipoprotein), DM (Diabetes Mellitus), LDL (Low-Density Lipoprotein).

The aggregated analysis of variable frequency, conducted from the rankings provided by sixteen studies [14,15,16,17,18,19,20,21,22,23,24,25,26,27,28,34], allowed for the identification of a set of predictors for the AI-based diagnosis of liver fibrosis. Age and AST stood out as the most consistently frequent variable overall, closely followed by a group of hepatic and metabolic markers (Figure 3). This ranking reflects feature recurrence across studies and should not be interpreted as a measure of individual predictive importance or causal association with fibrosis.

## 4. Discussion

This systematic review demonstrates that AI models based on clinical, laboratory, and imaging data offer new and promising opportunities for a more accurate staging of liver fibrosis. These models outperform traditional non-invasive methods, such as FIB-4, and achieve an AUROC close to or exceeding 0.9 in a significant proportion of the studies. Furthermore, based on the calculated score of the most frequent features, the ranking of the top four variables reflects the basis of already established non-invasive scores, such as FIB-4 (which uses AST, ALT, platelets, and age).

Deep learning models require large datasets; however, several studies used relatively small cohorts, often including only a few hundred images or patients [18,20,24,25,31,32,33]. Combining image analysis with clinical and laboratory data may improve model robustness. For example, the ALADDIN study reported higher diagnostic performance when laboratory data were integrated with Vibration-Controlled Transient Elastography (VCTE) (AUC 0.791 vs. 0.745 for VCTE alone) [14]. To address limited sample sizes, some studies employed *k*-fold cross-validation [31,33]; however, this strategy may underestimate true error and performance variability, particularly in datasets with limited heterogeneity. This limitation becomes more pronounced in high-capacity AI models with large numbers of trainable parameters, such as ensemble methods and deep neural networks, where repeated reuse of small datasets across folds increases the risk of overfitting and optimistic bias. In such scenarios, cross-validation may inadvertently favor complex models that exploit cohort-specific patterns rather than clinically meaningful features. By contrast, the hold-out approach, which enforces a strict separation between training and testing/validation datasets, offers a more conservative and clinically realistic estimate of model generalizability [35].

Despite promising results, external validation remains essential in this AI field. Most studies evaluated their models only on internal cohorts, and few have been implemented in clinical practice, which hinders the generalization of their results to broader and more diverse populations [34].

Another major limitation concerns the accuracy of fibrosis staging. Most studies dichotomize fibrosis severity using thresholds (e.g., ≥F2 or ≥F3) rather than determining the exact stage (e.g., F2 vs. F3). This approach prevents investigators from knowing their precise fibrosis level, reducing the precision of therapeutic decisions and prognostic assessment.

A further challenge involves the classification of intermediate stages (F2 and F3). These stages are characterized by more subtle and heterogeneous histological changes, which are inherently more difficult for algorithms to detect. Consequently, some studies simplify the classification to advanced categories (e.g., ≥F3 or F4), where histopathological features, such as cirrhosis, are more readily recognized by AI models [11].

In addition, most studies focus on assessing fibrosis stages ≥F2. However, the ability to reliably identify stage F1 would be highly beneficial, as early detection of liver fibrosis enables timely therapeutic intervention and increases the likelihood of liver parenchyma regeneration. One major challenge in detecting early fibrosis is the histological similarity between mild fibrotic tissue and normal parenchyma [11].

### 4.1. AI-Assisted Imaging and Radiomics for Liver Fibrosis: A Complementary Perspective

In addition to performance gains, imaging-based AI models offer unique advantages in liver disease assessment by capturing spatial and morphological features associated with fibrosis progression that are inaccessible to conventional clinical scores. Deep learning approaches applied to histopathology and radiology images enable objective quantification of morphological features, which are central to fibrosis staging. Although CT and MRI-based opportunistic screening and radiomics studies were identified during the literature search, they were excluded from the systematic analysis because they did not specifically address MASLD, in accordance with the predefined inclusion criteria. Nevertheless, these studies provide important contextual insights into the expanding role of AI-assisted imaging in liver fibrosis assessment and are therefore discussed herein. Deep-learning and radiomic studies using CT, MRI and ultrasound have demonstrated the ability to extract high-dimensional spatial and textural features reflecting hepatic fibrosis progression that are not captured by conventional laboratory-based scores [36,37,38,39,40,41].

A representative example of this paradigm is the quantification of liver surface nodularity (LSN) on routine CT and MRI. A systematic review and meta-analysis demonstrated that the LSN score achieves robust diagnostic performance for hepatic fibrosis, with pooled AUCs of 0.90 for significant fibrosis, 0.89 for advanced fibrosis, and 0.87 for cirrhosis, as well as a sensitivity of up to 88% for advanced fibrosis, despite substantial inter-study heterogeneity. Importantly, LSN provides an objective morphological biomarker of hepatic architectural remodeling that can be extracted from standard cross-sectional imaging without additional acquisition or specialized equipment, making it particularly suitable for opportunistic fibrosis screening in large imaging cohorts [42].

These imaging-derived biomarkers offer a non-invasive and quantitative framework for detecting subclinical fibrosis and for opportunistic screening in patients undergoing abdominal imaging for unrelated indications. Importantly, the integration of imaging features with clinical and laboratory data represents a major advantage of AI-assisted diagnosis. Multimodal models combining radiomics with biochemical markers and demographic variables have shown superior diagnostic performance compared with unimodal approaches [40], supporting a holistic view of fibrosis as a systemic and spatially heterogeneous disease. This fusion strategy is particularly promising for MASLD, where metabolic, inflammatory, and structural changes evolve concurrently. Although robust MASLD-specific radiomics cohorts remain limited, these imaging-based frameworks establish a methodological foundation for future MASLD-focused research.

### 4.2. Study Transparency

A cross-study assessment reveals limited transparency in how features were selected to train the AI models. In several reports, it is unclear whether variable selection was performed prior to the train–validation–test split or whether the selection process inadvertently incorporated information from the validation or test sets. This distinction is critical: when feature selection is influenced by data outside the training set, the model gains access to information it should not observe, characterizing data leakage. Such leakage systematically inflates performance estimates, producing overly optimistic AUROC values and reducing the credibility and real-world generalizability of the models [43].

By contrast, the feature-frequency approach adopted in our review provides an additional layer of interpretability and may support future studies by identifying which variables consistently appear across independent models. This aggregated analysis highlights biomarkers that are already well established in hepatology—such as Aspartate Aminotransferase (AST), Alanine Aminotransferase (ALT), platelet count, and age—reinforcing their pathophysiological relevance and demonstrating that the most predictive features selected by AI align with traditional clinical knowledge. Consequently, this method not only mitigates the lack of transparency observed in individual studies but also offers a reproducible, data-driven framework to guide feature selection in future AI model development.

Some studies do not make the feature selection method clear [22], whether it was based on clinical knowledge or a statistical method, such as LASSO (Least Absolute Shrinkage and Selection Operator) [17]. This knowledge is important because if the variables were chosen based on medical knowledge, the model tends to better reflect clinical practice and avoids including irrelevant data or data without a pathophysiological relationship [44].

Some studies do not clearly report the exact number of features used. Alkhouri et al. [14] and Suárez et al. [25] even present varying feature counts within their reports. Despite this reporting issue, the feature-to-sample ratio was generally acceptable across studies. The “curse of dimensionality” warns that adding features exponentially expands the search space, increasing sparsity and overfitting risk; to mitigate this, Berisha et al. recommend at least 10–20 samples per feature. When the number of features approaches or exceeds the sample size, models become unstable and less generalizable, and their clinical applicability is compromised [45].

Some studies lacked transparency regarding methodological details, particularly in describing the AI model architecture. For instance, Suárez et al. [24,25] did not report the absolute number of patients in training, validation, and testing, providing only percentages, which complicates replicability and increases the risk of sampling bias. Similarly, studies using biopsy or elastography as the gold standard [15,19,25] often failed to specify the exact number of patients undergoing each procedure, limiting the assessment of robustness and comparability with other works.

Another important point is that some studies did not compare the performance of their AI with other established non-invasive methods, such as FIB-4 or the NAFLD Fibrosis Score (NFS) [18,19,24,25,28]. This absence of comparative analysis restricts the understanding of the true incremental value that AI models offer in relation to the traditional tools already available in clinical practice.

### 4.3. Limitations of This Study

This review has limitations, the most important being the high heterogeneity among the included studies and the limited number of external validations. Differences in patient cohorts, imaging modalities, AI algorithms, and reference standards highlight the need for greater standardization in the field and suggest that, at present, results should be interpreted on a technique-by-technique basis. For future research, it is imperative that new AI models undergo external validation in multicenter and multiethnic cohorts. The development of larger databases, the standardization of analytic methodologies, and the deeper integration of clinical and imaging variables are essential steps to enable AI systems to achieve reliable, reproducible, and clinically actionable performance in the non-invasive diagnosis of liver fibrosis.

### 4.4. Implementation Feasibility of AI Models in Clinical Practice

The clinical impact of artificial intelligence for liver fibrosis remains contingent upon their feasibility of implementation. Most studies focused primarily on algorithmic development and validation, while providing limited evidence of the technical infrastructure required for deployment, integration into clinical workflows, or long-term operational sustainability. In practice, successful implementation demands interoperability with electronic health records, laboratory information systems, and radiology platforms, as well as secure data pipelines compliant with deployment and data protection regulations. Notably, the majority of models in this review were trained on clinical and laboratory variables, which substantially lowers the technical barrier to adoption compared with image-based deep learning systems, as these models can operate within minimal software integration [46].

The applicability of AI-assisted diagnosis also varies across healthcare settings. In primary and secondary care environments, lightweight models based on routinely available clinical and biochemical data may serve as effective population-level screening tools, enabling early identification of patients at high risk for significant fibrosis and optimizing referrals to specialized centers. In contrast, tertiary hospitals and academic centers are better positioned to implement more complex deep learning pipelines that incorporate imaging data, such as histopathology, CT, MRI or elastography, due to greater availability of computational infrastructure and specialized personnel. This tiered adoption framework highlights the potential for scalable deployment of AI solutions, tailored to the resources and clinical demands of each level of care [47].

Physician acceptance represents another critical determinant of clinical translation. Concerns regarding model interpretability and workflow disruption may limit adoption if not adequately addressed. Structured user training, transparent reporting of model limitations, and the incorporation of explainability tools are essential to foster trust and promote effective human–AI collaboration. Importantly, AI systems should be positioned as clinical decision support tools rather than autonomous diagnostic instruments, reinforcing the physician’s central role in patient management and reducing resistance to adoption [48].

Finally, cost-effectiveness and regulatory approval constitute fundamental barriers and opportunities. Although formal economic analyses were not reported in the reviewed studies, AI models that rely solely on laboratory and clinical data offer a favorable cost profile, with minimal marginal cost per patient after deployment and significant potential to reduce unnecessary elastography, invasive biopsies, and late-stage disease complications. Addressing these translational challenges is essential for optimal MASLD outcomes [49].

Although AI models consistently demonstrated superior diagnostic accuracy compared with traditional non-invasive scores, the reviewed studies did not evaluate how AI-assisted diagnosis modifies clinical decision-making, patient management, quality of life, or long-term prognosis. This absence of outcome-driven evidence substantially limits the current clinical value of these tools and helps explain why, despite promising performance, AI-based fibrosis models have not yet been incorporated into major clinical guidelines. In addition to this lack of clinical impact assessment, several practical barriers hinder adoption, including limited external validation, insufficient generalizability, poor model transparency, and difficulties in integrating AI systems into routine clinical workflows. Future research must therefore move beyond retrospective accuracy and focus on prospective studies that demonstrate tangible benefits for patient outcomes, healthcare resource utilization, and cost-effectiveness, which are essential prerequisites for guideline endorsement and real-world implementation [50].

## 5. Conclusions

This systematic review demonstrates that AI has reached a high level of diagnostic performance for the non-invasive assessment of liver fibrosis in patients with MASLD, consistently outperforming widely used clinical scores such as FIB-4 and NFS. The convergence of evidence from clinical, laboratory, and imaging-based models indicates that AI can substantially improve fibrosis risk stratification and early detection. However, the clinical translation of these models remains constrained by limited external validation, high methodological heterogeneity, and the absence of prospective studies evaluating the impact of AI-assisted diagnosis on patient management, clinical outcomes, and healthcare resource utilization. Future research should prioritize large, multicenter prospective studies and standardized reporting of AI development and validation to establish the real-world clinical value of these technologies.

## Figures and Tables

**Table 1:** Inclusion and exclusion criteria for the studies.

Parameters	Inclusion Criteria	Exclusion Criteria
Population	Patients ≥ 18 years old diagnosed with MASLD	Studies in animal models, in vitro studies, pediatric populations, or other liver diseases
Intervention	Applications of artificial intelligence (AI), such as machine learning, deep learning, or neural networks, for the assessment or prediction of liver fibrosis	Studies that do not use AI tools as a central part of the analysis
Comparator	Studies with or without a comparator group. When present, comparisons with traditional methods (e.g., elastography, biopsy, clinical scores)	Studies that only describe imaging methods or laboratory tests without a link to AI
Outcome	Performance of AI in identifying, classifying, or predicting the degree of liver fibrosis; diagnostic accuracy; sensitivity/specificity; AUROC	Studies without analysis of clinical or predictive outcomes, or that do not report AI performance metrics
Study Type	Original articles from retrospective or prospective studies, cohorts, validation studies, or cross-sectional studies with real clinical data (medical records, imaging, histology, laboratory tests) used in AI models	Abstracts, systematic reviews, editorials, letters to the editor, study protocols, commentaries, or opinion articles based exclusively on theoretical simulations
Performance Assessment	Presence of quantitative metrics such as AUROC, sensitivity, specificity, and accuracy	Studies that do not present any objective performance metrics
Study Language	English	Languages other than English
Data Splitting	Studies that applied data splitting into training/testing/validation or train/validation sets	Studies without a clear description of the model validation methodology
Gold Standard	Studies that used accepted methods for fibrosis diagnosis as a reference (e.g., liver biopsy, elastography)	Studies without a clear definition of the gold standard to validate the model’s results
Features	Presented the features used (e.g., clinical, laboratory, histological, radiological, or combined data) as input variables in the AI models	Studies that did not clearly describe the data used. Lack of transparency in the feature selection method, excluding studies that used the test set in the feature selection process, eliminating the risk of the curse of dimensionality with a minimum ~10:1 ratio

Abbreviation: AUROC (Area Under the Receiver Operating Characteristic curve).

**Table 2:** Characteristics of studies developing AI models from clinical and laboratory data (predominantly machine learning, with limited neural networks).

Reference	Sample Size (*n*) Patients	Data Splitting *	Validation	Gold Standard	Fibrosis Classification	AI ** Architecture	AI Performance (AUROC)	Traditional NIT Performance (AUROC)
Alkhouri N et al. (2022) [14]	3630	827/1504/1299	External	Biopsy	≥F2	*RF*, *GBM*, *XGB*	0.717 (Ensemble); 0.683 (XGB); 0.678 (GBM); 0.688 (RF)	0.655 (FIB-4); 0.632 (Liver Risk)
Dabbah S et al. (2025) [15]	1158	618/540	Internal	Elastography or Biopsy	≥F3 or ≥9.3 kPa	*XGB*, *LR*, **ANN**, *SVM*, *RF*	0.91 (XGB); 0.89 (LR); 0.89 (ANN); 0.89 (SVM); 0.90 (RF)	0.78 (FIB-4); 0.81 (NFS)
Fan R et al. (2024) [16]	828	703/125	External	Biopsy	F3 or F4	*RF*, *LR*, *XGB*, *NB*, *KNN*, *SVM*, *Bagging*	0.808–0.964 (F3); 0.718–0.985 (F4)	0.795 (FIB-4 for F3); 0.857 (FIB-4 for F4)
Feng G et al. (2021) [17]	553	278/275	Internal	Biopsy	≥F2	*RF*, *LR*	0.893 (RF); 0.786 (LR)	0.578 (FIB-4)
Ginter-Matuszewska B et al. (2025) [18]	178	153/25	Internal	Elastography	≥F2 or >7.0 kPa	*LR*, *DT*	0.656 (LR); 0.622 (DT)	N
Hassoun S et al. (2024) [19]	6082	5962/120	External	Elastography or Biopsy	≥F3	*NAIF*	0.83	N
Lu CH et al. (2024) [20]	194	135/59	Internal	Biopsy	≥F2	*SVM*, *RF*, *KNN*, *XGB*, *LR*	0.770 (XGB); 0.768 (SVM); 0.748 (LR); 0.712 (KNN); 0.738 (RF)	0.710 (FIB-4)
Mamandipoor B et al. (2023) [21]	1151	808/343	Internal	Elastography	>8 kPa	*XGB*, **FF**, *LR*	0.71 (XGB); 0.70 (LR); 0.74 (FF)	0.61 (FIB-4)
Okanoue T et al. (2021) [22]	434	324/110	Internal	Biopsy	≥F1, ≥F2 or ≥F3	**NN**	0.922 (≥F1); 0.901 (≥F2); 0.911 (≥F3)	0.766 (FIB-4 for ≥F1); 0.809 (FIB-4 for ≥F2); 0.771 (FIB-4 for ≥F3)
Sang C et al. (2021) [23]	784	540/244	Internal	Biopsy	≥F3	*LR*	0.89	0.85 (FIB-4)
Suárez M et al. (2023) [24]	215	150/65	Internal	Biopsy	≥F3	*XGB*, *SVM*, *DT*, *GNB*, *KNN*	0.95 (XGB); 0.91 (KNN); 0.84 (GNB); 0.88 (DT); 0.87 (SVM)	N
Suárez M et al. (2023) [25]	211	148/63	Internal	Biopsy or Elastography	≥F2	*XGB*, *SVM*, *BLDA*, *LR*, *DT*, *KNN*	0.92 (XBG); 0.82 (SVM); 0.79 (BLDA); 0.75 (LR); 0.83 (DT); 0.84 (KNN)	N
Verma N et al. (2024) [26]	1656	1153/283/220	Internal	Biopsy	≥F2	*RF*, *XGB*	0.714 (RF); 0.764 (XBG)	0.699 (FIB-4)
Xiong FX et al. (2025) [27]	746	522/224	Internal	Biopsy	≥F3	*XGB*, *RF*, *SVM*, *LR*, *NB*	0.917 (XGB); 0.840 (RF); 0.740 (SVM); 0.790 (LR); 0.503 (NB)	0.752 (FIB-4)
Yamaguchi K et al. (2023) [28]	1198	898/300	Internal	Biopsy	≥F3	**NN**	0.976	N

* Train/Test/Validation or Train/Validation. ** Deep-learning algorithms are highlighted in **bold** and machine-learning algorithms are highlighted in *italic*. Abbreviation: RF (Random Forest), GBM (Gradient Boosting Machines), XGB (Extreme Gradient Boosting or XGBoost), LR (Logistic Regression), ANN (Artificial Neural Network), SVM (Support Vector Machine), DT (Decision Tree), KNN (K-Nearest Neighbors), NB (Naive Bayes), NAIF (NAFLD Artificial Intelligence Fibrosis model), FF (Feed-Forward Neural Network), NN (Neural Network), GNB (Gaussian Naive Bayes), BLDA (Bayesian Linear Discriminant Analysis), N (No), Bagging (Bootstrap Aggregating).

**Table 3:** Characteristics of imaging-based deep learning studies (radiology/histology/elastography).

Reference	Sample Size (*n*)	Data Splitting *	Validation	Gold Standard	Fibrosis Classification	AI Architecture	Image Type	AI Performance (AUROC)
Cunha GM et al. (2022) [29]	2319 images	1761/558	External	Biopsy	≥F1, ≥F2, ≥F3, =F4	U-Net	Magnetic Resonance Elastography (MRE)	0.89 (≥F1); 0.92 (≥F2); 0.92 (≥F3); 0.93 (=F4)
Lu X et al. (2024) [30]	1083 images	707/209/167	Internal	Elastography	≥7 kPa	DenseNet-201	Tongue photographs	0.893
Naik SN et al. (2023) [31]	152 images	**5-fold cross-validation** **(70-20-10%)**	Internal	Biopsy	>F2	ResNet-18	Histological	0.87
Preechathammawong N et al. (2024) [32]	176 images	(**)/146/30	Internal	Biopsy	F0, F1, F2, F3, F4	U-Net	Histological	*** 80.82% (F0–F1 and F2–F4); 89.73% (F0–F2 and F3–F4)
Yin C et al. (2024) [33]	132 images	**3-fold-cross-validation**	Internal	Biopsy	≥F1, ≥F2, ≥F3, ≥F4	PDGCN	Histological	0.83 (≥F1); 0.78 (≥F2); 0.86 (≥F3); 0.88 (≥F4)
Zhan H et al. (2023) [34]	1375 images and 203 patients	143/60	External	Biopsy	F0, F1, F2, F3–F4	VGG16	Histological	0.99 (F0); 0.83 (F1); 0.80 (F2); 0.90 (F3–F4)

* Train/Test/Validation or Train/Validation. The use of the *k*-fold cross-validation method is highlighted in **bold**. ** Preechathammawong N et al. [32] used a Pre-trained AI. Therefore, information about the training set is not available. *** Agreement reported instead of AUROC. Abbreviation: U-Net (U-shaped Convolutional Neural Network), DenseNet-201 (Densely Connected Convolutional Network-201), ResNet-18 (Residual Neural Network-18), VGG16 (Visual Geometry Group-16), PDGCN (Primal-Dual Graph Convolution Networks).

## Data Availability

The original contributions presented in this study are included in the article/Supplementary Materials. Further inquiries can be directed to the corresponding author.

## Supplementary Materials

The following supporting information can be downloaded at: https://www.mdpi.com/article/10.3390/diagnostics16020261/s1, Supplementary Table S1: PRISMA 2020 Checklist, Supplementary Table S2: Boolean Search Strings Structure, Supplementary Table S3: Risk of Bias Assessment of the Studies using the Quality Assessment of Diagnostic Accuracy Studies (QUADAS-2) Tool. Reference [51] is cited in the supplementary materials.

## Abbreviations

The following abbreviations are used in this manuscript:

AI	Artificial Intelligence
ALT	Alanine Aminotransferase
ANN	Artificial Neural Network
AST	Aspartate Aminotransferase
AUROC (AUC)	Area Under the Receiver Operating Characteristic Curve
BLDA	Bayesian Linear Discriminant Analysis
BMI	Body Mass Index
CNN	Convolutional Neural Network
DT	Decision Tree
FF	Feed-Forward Neural Network
FIB-4	Fibrosis-4 Index
GBM	Gradient Boosting Machine
GGT	Gamma-Glutamyl Transferase
GNB	Gaussian Naive Bayes
HDL	High-Density Lipoprotein
KNN	K-Nearest Neighbors
LASSO	Least Absolute Shrinkage and Selection Operator
LR	Logistic Regression
LSN	Liver Surface Nodularity
MASH	Metabolic-Dysfunction-Associated Steatohepatitis
MASLD	Metabolic-Dysfunction-Associated Steatotic Liver Disease
MINIMAR	Minimum Information for Medical AI Reporting
MRE	Magnetic Resonance Elastography
NAIF	NAFLD Artificial Intelligence Fibrosis Model
NB	Naive Bayes
NFS	NAFLD Fibrosis Score
NITs	Non-Invasive Tests
NN	Neural Network
PDGCN	Primal-Dual Graph Convolution Networks
PRISMA	Preferred Reporting Items for Systematic Reviews and Meta-Analyses
QUADAS-2	Quality Assessment of Diagnostic Accuracy Studies, version 2
RF	Random Forest
SAFE	Steatosis-Associated Fibrosis Estimator
SR	Systematic Review
SVM	Support Vector Machine
T2DM	Type 2 Diabetes Mellitus
VCTE	Vibration-Controlled Transient Elastography
VGG16	Visual Geometry Group 16
XGBoost (XGB)	Extreme Gradient Boosting
